# Leveraging Analytics to Shape California’s Caregiver Equity Roadmap

**DOI:** 10.1093/geroni/igaf122.998

**Published:** 2025-12-31

**Authors:** Shikha Bhurtel, Rachael Fulp-Cooke, Heather Young, Janice Bell

**Affiliations:** University of California, Davis, Davis, California, United States; Betty Irene Moore School Of Nursing at UC Davis, Sacramento, California, United States; Betty Irene Moore School Of Nursing at UC Davis, Sacramento, California, United States; Betty Irene Moore School Of Nursing at UC Davis, Sacramento, California, United States

## Abstract

Shifts in population and other factors are reshaping caregiving in California. To ensure access and effective support, publicly funded caregiver services and supports must be responsive to population trends and caregiver characteristics. The California Department of Aging (CDA) partnered with the Family Caregiving Institute (FCI) at UC Davis Betty Irene Moore School of Nursing to develop a roadmap to outline strategies and actions to improve publicly funded caregiver support over five years. This study identified data sources and analytical approaches to develop the California Caregiver Equity Roadmap (“Roadmap”). Data sources included administrative records from publicly funded caregiver support programs, caregiver assessments from the California Caregiver Resource Centers, and population-based surveys. Analyses integrated these data sources to examine population trends and caregiver characteristics. Key sociodemographic and health characteristics included age distribution, geographic patterns, race and ethnicity, poverty level, broadband access, care dependency ratios, care receiver needs, and attributes of the caregiving role. Analyzing the intersection of population trends and caregiver characteristics provided a foundation for the Roadmap’s development. The findings provide a deeper understanding of caregiving in California, insights for improving services and supports across the state, and offer a framework for other states to follow. Research implications include using data to better understand caregiver needs, improve service delivery, and guide policy decisions and program development.

